# Metabolic dysfunction–associated steatotic liver disease in Cushing’s syndrome: prevalence, determinants, and changes after remission

**DOI:** 10.3389/fendo.2026.1790066

**Published:** 2026-04-24

**Authors:** Enes Ucgul, Burak Menekse, Revah Cankaya, Murat Bugra Gorgulu, Bekir Ucan, Erman Cakal, Muhammed Kizilgul

**Affiliations:** 1 Department of Endocrinology and Metabolism, Ankara Etlik City Hospital, Ankara, Türkiye; 2 Division of Diabetes, Endocrinology and Metabolism Department of Medicine, University of Minnesota, Minneapolis, MN, United States

**Keywords:** Cushing’s syndrome, fibrosis risk, hepatosteatosis, hypercortisolism, metabolic dysfunction–associated steatotic liver disease

## Abstract

**Background:**

Metabolic dysfunction–associated steatotic liver disease (MASLD) is increasingly recognized as a systemic manifestation of endocrine disorders. Cushing’s syndrome (CS), characterized by chronic endogenous hypercortisolism, is associated with profound metabolic disturbances that may predispose to MASLD; however, data on its prevalence, determinants, and reversibility after remission remain limited and inconsistent.

**Objective:**

To evaluate the prevalence of MASLD in patients with CS, assess changes in hepatic steatosis and fibrosis-related indices following biochemical remission, and identify independent clinical, metabolic, and etiological predictors of MASLD.

**Methods:**

In this retrospective study, 126 adult patients with endogenous CS diagnosed between 2014 and 2025 were included. MASLD was defined according to contemporary consensus criteria. Imaging-based hepatic steatosis was evaluated by abdominal ultrasonography. Hepatic steatosis and fibrosis risk were assessed using the hepatic steatosis index (HSI) and Fibrosis-4 index (FIB-4), respectively, during the active disease phase and six months after biochemical remission. Comparisons were performed between adrenal and pituitary CS. Logistic regression analyses were used to identify factors independently associated with MASLD.

**Results:**

MASLD was present in 62.7% of patients with CS. At baseline, MASLD prevalence did not differ significantly between adrenal and pituitary CS. HSI decreased from the active phase to six months after remission in both adrenal and pituitary CS (p < 0.01 for both), indicating partial reversibility of hepatosteatosis following cortisol normalization. Overall, FIB-4 values did not change; however, patients with elevated baseline fibrosis risk (FIB-4 ≥1.3) demonstrated a significant reduction after remission (p = 0.010). In multivariate analysis, higher body mass index (adjusted OR 1.30), longer duration of symptoms before diagnosis (adjusted OR 1.06), and adrenal CS (adjusted OR 2.90) were independently associated with MASLD, whereas increasing age was inversely associated. Biochemical measures of cortisol excess were not independently related to MASLD.

**Conclusions:**

MASLD is highly prevalent in CS and is primarily driven by metabolic burden, disease chronicity, and etiology rather than by biochemical severity of hypercortisolism. Hepatic steatosis shows partial improvement after remission, whereas the risk of fibrosis declines primarily among patients with elevated baseline risk. These findings support routine hepatic assessment in CS, particularly those with prolonged disease duration and increased metabolic risk.

## 1 Introduction

Metabolic dysfunction–associated steatotic liver disease (MASLD) is among the most common causes of chronic liver disease, with prevalence increasing over time (1, 2). The pathogenesis of MASLD is multifactorial and closely linked to metabolic dysregulation, including diabetes mellitus, hypothyroidism, obesity, hypogonadism, and Cushing’s syndrome (CS) (3).

CS is a rare endocrine disorder characterized by sustained endogenous hypercortisolism and is associated with profound metabolic derangements, including insulin resistance, central obesity, and dyslipidemia—key drivers in the development of MASLD (4). Glucocorticoids (GCs) exert complex effects on hepatic lipid metabolism and adipose tissue distribution through glucocorticoid receptor–mediated transcriptional regulation and enhanced local cortisol regeneration via 11β-hydroxysteroid dehydrogenase type 1 (11β-HSD1). These mechanisms increase hepatic free fatty acid flux and promote steatogenesis. Conversely, GCs suppress NF-κB– and AP-1–mediated inflammatory signaling pathways (e.g., IL-6 and TNF-α) and modulate profibrotic signaling (e.g., TGF-β), potentially attenuating intrahepatic inflammation and influencing fibrogenesis. Together, these opposing effects may contribute to the heterogeneous and paradoxical hepatic phenotype observed in CS (5–7).

Previous studies evaluating the prevalence of MASLD in CS have yielded inconsistent results. Some cohorts have reported prevalence rates comparable to those in the general population (8), whereas others have demonstrated a substantially higher burden of MASLD among patients with CS (9). This variability likely reflects heterogeneity in study populations, diagnostic criteria, and methodologies used to assess hepatic steatosis. Furthermore, evidence comparing the burden of MASLD across etiological subtypes of CS, particularly adrenal versus pituitary disease, remains limited (10, 11). Similarly, studies examining associations between standard diagnostic cortisol assessments in CS and MASLD are sparse and report conflicting findings (12). Noninvasive indices such as the hepatic steatosis index (HSI) and FIB-4 score are widely used to estimate hepatic steatosis and fibrosis, respectively, and may provide practical tools for longitudinal assessment of liver involvement in patients with CS (13, 14).

This study aimed to evaluate the prevalence of MASLD in patients with CS, examine changes in hepatosteatosis and fibrosis-related indices (HSI and FIB-4) between the active disease phase and six months after biochemical remission, and identify the independent metabolic, clinical, and etiological determinants associated with MASLD.

## 2 Materials and methods

### 2.1 Study design and population

This retrospective cohort study included patients diagnosed with CS between January 2014 and April 2025 at Ankara Etlik City Hospital and Dışkapı Yıldırım Beyazıt Training and Research Hospital. The diagnosis of CS was established based on clinical features, biochemical confirmation, and radiological findings in accordance with current international guidelines (15).

**Figure 1:**
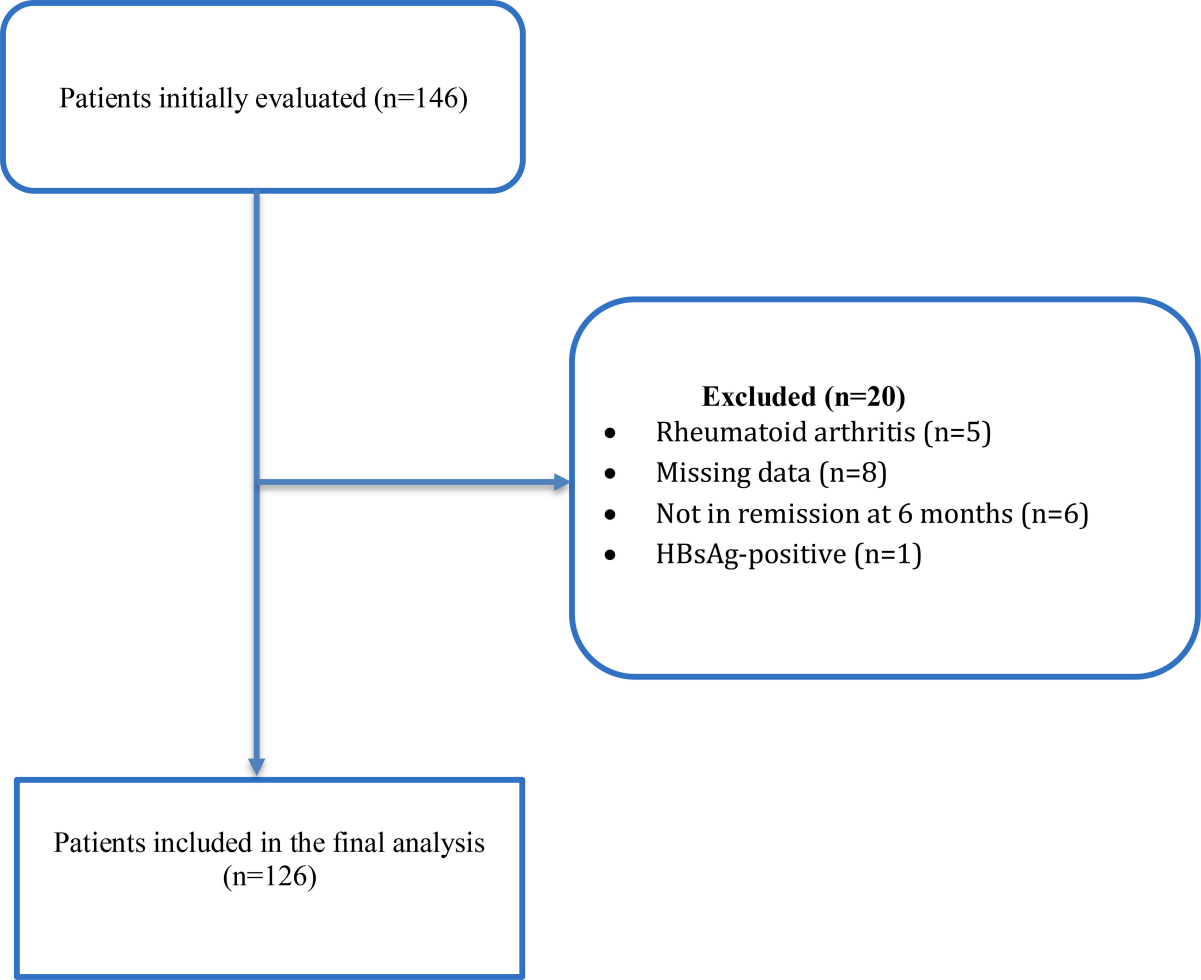
Patient enrollment and exclusion flowchart.

Patients aged ≥18 years with confirmed endogenous hypercortisolism, available baseline abdominal ultrasonography (USG), and complete baseline biochemical evaluation were eligible for inclusion. Patients were excluded if they had incomplete medical records, missing key diagnostic data (including midnight serum cortisol, late-night salivary cortisol, 1-mg or 2-mg dexamethasone suppression test (DST) results, or 24-hour urinary free cortisol measurements), or lacked baseline liver-related laboratory parameters required for index calculations. Additional exclusion criteria included pre-existing chronic liver disease unrelated to metabolic dysfunction, alcohol-related liver disease, viral hepatitis, active malignancy, pregnancy, or use of medications known to significantly affect hepatic steatosis or fibrosis indices. After applying these criteria, 126 patients were included in the final analysis (**Figure 1**).

### 2.2 Clinical and biochemical assessment

Demographic characteristics, body mass index (BMI), comorbidities (including diabetes mellitus and hypertension), and duration of symptoms prior to diagnosis were extracted from medical records. Biochemical evaluation included midnight serum cortisol, late-night salivary cortisol, 24-hour urinary free cortisol (UFC), dehydroepiandrosterone sulfate (DHEA-S), and cortisol levels following 1-mg and 2-mg dexamethasone suppression tests. Furthermore, postoperative remission was defined as early postoperative hypocortisolism, characterized by a morning serum cortisol level <5 µg/dL in the early postoperative phase. The need for glucocorticoid replacement therapy due to adrenal insufficiency was considered supportive evidence of successful surgical remission. MASLD was defined according to contemporary consensus criteria, based on the presence of hepatic steatosis together with metabolic risk factors (16).

### 2.3 Assessment of hepatic steatosis and fibrosis

Hepatic steatosis was evaluated by abdominal USG, and steatosis risk was additionally assessed using the HSI (17), calculated as follows:


HSI = 8 × (ALT/AST) + BMI (+ if female) (+ if diabetes mellitus present)

where ALT and AST represent alanine aminotransferase and aspartate aminotransferase levels (U/L), respectively, and BMI is expressed in kg/m². Diabetes mellitus and female sex were included as dichotomous variables, in line with the original index definition. Furthermore, for hepatic steatosis, the optimal HSI cut-off value was determined using ROC curve analysis in patients diagnosed with steatosis on ultrasonography. The optimal threshold was identified using the Youden index.

Hepatic fibrosis risk was evaluated using the Fibrosis-4 index (FIB-4) (18), calculated using the standard equation:


$$\text{FIB}-4\;=\;\left[\text{Age}\;\left(\text{years}\right)\;\times \;\text{AST}\;\left(\text{U}/\text{L}\right)\right]/\left[\text{Platelet}\;\text{count}\;\left(10^{9}/L\right)\;\times \;\surd \text{ALT }\left(\text{U}/\text{L}\right)\right].$$

Additionally, for assessing liver fibrosis, the widely accepted FIB-4 cut-off of **1.3** was used to indicate an increased risk of significant fibrosis.

Both indices were calculated at two predefined time points:

- Active phase of Cushing’s syndrome (baseline, prior to definitive treatment).
- Six months after biochemical remission.

Changes in HSI and FIB-4 values between the active disease phase and the remission period (at six months) were used to assess the evolution of hepatic steatosis and the risk of fibrosis after cortisol normalization.

### 2.4 Ethical approval

The study was conducted in accordance with the Declaration of Helsinki and approved by the local Ethics Committee (approval number/date: AEŞH-BADEK2-2025-112/20-05-2025). The institutional review board waived the requirement for informed consent due to the study’s retrospective design.

### 2.5 Statistical analysis

Continuous variables were tested for normality and are presented as median (interquartile range). Categorical variables are expressed as counts and percentages. Comparisons between adrenal and pituitary CS were performed using the Mann–Whitney U test for continuous variables and the chi-square or Fisher’s exact test for categorical variables. Within-group comparisons of HSI and FIB-4 between active disease and remission were conducted using paired non-parametric tests. To identify factors associated with MASLD, univariate logistic regression analyses were initially performed. Variables with clinical relevance or a p-value <0.20 in univariate analysis were entered into a multivariate logistic regression model. Results are presented as odds ratios (ORs) with 95% confidence intervals (CIs). Statistical significance was defined as a two-sided p-value <0.05. All statistical analyses were conducted using SPSS software (version 27.0).

## 3 Results

### 3.1 Baseline characteristics according to Cushing’s syndrome etiology

Baseline demographic, clinical, and hormonal characteristics of patients with adrenal and pituitary CS are summarized in **Table 1**. The median age was comparable between the adrenal and pituitary groups (52 [44–62] vs. 50 [37–57] years, respectively), with no significant difference observed. A female predominance was observed in both groups, and the proportion of women did not differ significantly between adrenal and pituitary CS (82.6% vs. 89.5%; *p* = 0.273). Furthermore, Postoperative adrenal insufficiency was managed with physiologic glucocorticoid replacement therapy. The mean daily hydrocortisone replacement dose during follow-up was approximately 15–20 mg/day, administered in divided doses. The duration of steroid replacement therapy was significantly longer in patients with pituitary CS compared with those with adrenal CS (13.6 ± 5.2 vs. 9.8 ± 4.1 months, p = 0.01).

**Table 1:** Baseline demographic, clinical, and hormonal characteristics according to etiology.

Variable	Adrenal CS	Pituitary CS	p value
Age, years	52 (44-62)	50 (37-57)	0.134
Female sex, n (%)	57 (82.6%)	51 (89.5%)	0.273
Duration of steroid replacement (months)	9.8 ± 4.1	13.6 ± 5.2	0.01
BMI (kg/m²)	31 (29-33.6)	32.3 (29.5-35.4)	0.152
Diabetes mellitus, n (%)	32 (46.4%)	32 (56.1%)	0.275
Hypertension, n (%)	38 (66.7%)	54 (78.3%)	0.144
Duration of symptoms before diagnosis (months)	12 (8-14)	12 (7-15)	0.496
Midnight serum cortisol (µg/dL)	9.9 (7.52-14)	14.85 (11-17.35)	0.001
Late-night salivary cortisol (µg/dL)	0.2 (0.12-0.45)	0.4 (0.25-0.52)	0.001
DHEA-S (µg/dL)	28 (16-53)	150 (120-240)	0.001
1 mg DST result, µg/dL	7 (4.1-14)	9.3 (7-14)	0.032
2 mg DST result, µg/dL	6.9 (3.65-13)	6.3 (4.02-11.5)	0.453
24-UFC (µg/24h)	75 (50-120)	111(77-200)	0.009
MASLD (n/%)	45 (65.2%)	34 (59.6%)	0.520

Body Mass Index, BMI; DST, dexamethasone suppression test; DHEA-S, dehydroepiandrosterone sulfate; UFC, urinary free cortisol; MASLD, metabolic dysfunction–associated steatotic liver disease.

Reference ranges: normal 24-h UFC <45 µg/24 h; late-night salivary cortisol <0.42 µg/dL; midnight serum cortisol <7.5 µg/dL.

BMI was similar between groups, with median values of 31 (29–33.6) kg/m² in the adrenal group and 32.3 (29.5–35.4) kg/m² in the pituitary group (*p* = 0.152). The prevalence of diabetes mellitus (46.4% vs. 56.1%, *p* = 0.275), hypertension (66.7% vs. 78.3%, *p* = 0.144), and MASLD (65.2% vs. 59.6%, *p* = 0.520) did not differ significantly between the two etiological subgroups. Likewise, the duration of symptoms before diagnosis was comparable (12 [8–14] vs. 12 [7–15] months, *p* = 0.496).

In contrast, several hormonal parameters differed significantly according to etiology. Patients with pituitary CS exhibited higher midnight serum cortisol levels compared with those with adrenal CS (14.85 [11–17.35] vs. 9.9 [7.52–14] µg/dL, *p* = 0.001). Late-night salivary cortisol levels were also significantly higher in the pituitary group (0.4 [0.25–0.52] vs. 0.2 [0.12–0.45] µg/dL, *p* = 0.001). DHEA-S concentrations were markedly elevated in pituitary CS compared with adrenal CS (150 [120–240] vs. 28 [16–53] µg/dL, *p* = 0.001).

Cortisol levels after the 1-mg DST results were significantly higher in the pituitary group (9.3 [7–14] vs. 7 [4.1–14] µg/dL, *p* = 0.032), whereas cortisol levels after the 2-mg DST results did not differ between groups (*p* = 0.453). Additionally, 24-hour UFC levels were significantly higher in patients with pituitary CS than in those with adrenal CS (111 [77–200] vs. 75 [50–120]; *p* = 0.009).

Overall, as shown in **Table 2**, patients with MASLD had significantly higher BMI compared with those without MASLD in both adrenal CS and pituitary CS cohorts (Adrenal: 32 [30.2–36.1] vs 28.3 [27–31] kg/m², p<0.001; Pituitary: 33 [31–38] vs 30 [29–34.75] kg/m², p=0.010). Age tended to be lower in MASLD (+) patients in adrenal CS (52 [42–59] vs 55 [46.5–66] years), although this did not reach statistical significance (p=0.059), and no significant age difference was observed in pituitary CS (p=0.141). The proportion of female patients was comparable between the MASLD (+) and MASLD (−) groups in both cohorts (all p >0.05). Similarly, the prevalence of hypertension and diabetes mellitus did not differ significantly by MASLD status in adrenal or pituitary CS (all p>0.05). A greater proportion of MASLD-positive patients with pituitary CS had symptom duration ≥12 months (47.1% vs 17.4%, p = 0.021), whereas no significant difference was observed in adrenal CS (p = 0.468). Cortisol-related parameters, including 1-mg DST result, 24-h UFC, late-night salivary cortisol, and midnight serum cortisol, were comparable between MASLD groups across both etiological subtypes (all p > 0.05).

**Table 2:** Baseline characteristics of adrenal and pituitary CS patients according to MASLD status.

Variables	Adrenal CS			Pituitary CS		
	MASLD − (n=24)	MASLD + (n= 45)	p	MASLD − (n=23)	MASLD + (n= 34)	p
Demographics						
Age, years	55 (46.5-66)	52 (42-59)	0.059	52 (43-60)	45 (33.5-57)	0.141
Female sex, (%)	75%	86.7%	0.223	91.3%	88.2%	0.711
BMI, kg/m²	28.3 (27-31)	32 (30.2-36.1)	<0.001	30 (29-34.75)	33 (31-38)	0.010
Comorbidities						
Hypertension, (%)	79.2%	77.8%	0.894	56.5%	73.5%	0.181
Diabetes mellitus, (%)	41.7%	48.9%	0.567	55.6%	55.9%	0.962
Cushing-related variables						
Symptom duration (≥12 months, (%))	20.8%	28.9%	0.468	17.4%	47.1%	0.021
1 mg DST result, μg/dL	7.25 (4-17.75)	6.7 (4.3-14)	0.772	9.3 (7.2-14.7)	9.6 (7-14)	0.890
24-h UFC, µg/24h	74.5 (49-132.7)	75 (49.5-125)	0.845	110 (69-194)	112 (81-289)	0.255
Late-night salivary cortisol, μg/dL	0.2 (0.125-0.57)	0.21 (0.11-0.45)	0.566	0.37 (0.2-0.52)	0.40 (0.28-0.56)	0.726
Midnight serum cortisol, μg/dL	11.5 (7.6-14)	9.6 (6.6-12)	0.635	14.7 (9-20)	15 (11.5-16.75)	0.803
DHEA-S (µg/dL)	39.5 (14.5–53)	24 (23.5-96)	0.004	140 (120–232)	146 (104-166)	0.011
Metabolic profile						
HbA1c, %	5.7 (5.3-6.1)	5.8 (5.1-6.5)	0.807	5.8 (5-7.7)	6.5 (5.4-8.42)	0.632
LDL-C, mg/dL	119 (103-136)	124 (89-156)	0.751	122 (96-155)	125 (83-156)	0.897
HDL-C, mg/dL	47 (36-58)	47 (37-57)	0.887	49 (40-56)	45 (39-52)	0.379
TG, mg/dL	140 (101-161)	158 (125-236)	0.090	128 (78-224)	146 (100-262)	0.392
Liver biochemistry						
AST, U/L	18 (14-27.7)	18 (15-20.5)	0.649	14 (13-19)	17 (14-22.2)	0.133
ALT, U/L	20 (16.5-31.5)	24 (17-32)	0.416	16 (14-24)	24 (16-30.5)	0.023
GGT, U/L	20 (14.2-37)	23 (17-35.5)	0.673	16 (12-24)	22 (16.5-31)	0.046

ALT, alanine aminotransferase; AST, aspartate aminotransferase; BMI, body mass index; CS, Cushing syndrome; DST, dexamethasone suppression test; GGT, gamma-glutamyl transferase; HbA1c, glycated hemoglobin; HDL-C, high-density lipoprotein cholesterol; TG, Triglycerides; LDL-C, low-density lipoprotein cholesterol; MASLD, metabolic dysfunction–associated steatotic liver disease; UFC, urinary free cortisol; DHEA-S, dehydroepiandrosterone sulfate. *Reference ranges: normal 24-h UFC <45 µg/24 h; late-night salivary cortisol <0.42 µg/dL; midnight serum cortisol <7.5 µg/dL.*

### 3.2 HSI discrimination of ultrasonography-defined hepatic steatosis

**Figure 2:**
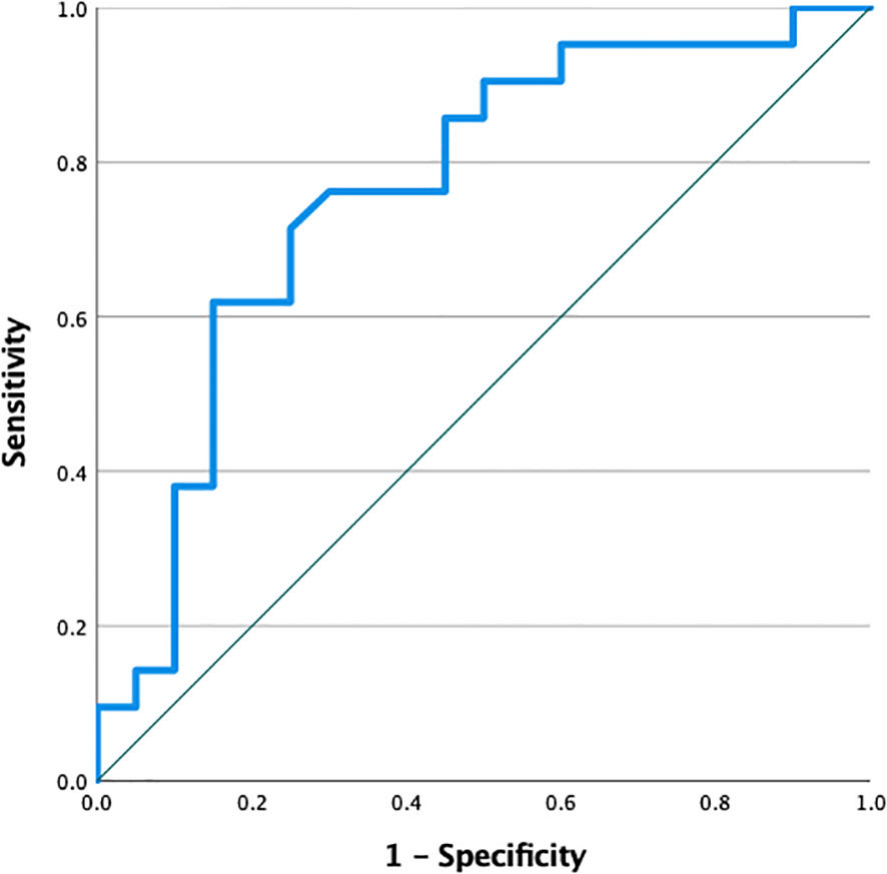
ROC curve of HSI for imaging-based hepatic steatosis assessed by abdominal USG.

ROC curve analysis was performed to evaluate the discriminative performance of the HSI for imaging-based assessment of hepatic steatosis using abdominal USG (**Figure 2**). HSI demonstrated acceptable discriminatory ability (AUC 0.763; 95% CI 0.612–0.914; p = 0.004) (**Figure 2**). The optimal HSI cutoff value was determined using the Youden index, and a threshold ≥43.5 was selected, yielding a sensitivity of 71.4% and a specificity of 75.0%.

### 3.3 Six-month changes in hepatic steatosis and fibrosis indices after remission

Changes in HSI over the six-month period following remission are presented in **Table 3**. During the active phase of disease, baseline HSI values were comparable between patients with adrenal and pituitary CS (43.33 [39.6–49.6] vs. 46.41 [41.9–51.1], respectively; *p* = 0.168). Similarly, HSI values measured at six months after remission did not differ significantly between the two etiological groups (42.9 [40.2–49.2] in adrenal CS vs. 44.51 [38.7–48.4] in pituitary CS; *p* = 0.273).

**Table 3:** Six-month change in HSI after remission.

Parameter	Adrenal CS	Pituitary CS	p-value
HSI – Active phase	43.33 (39.6-49.6)	46.41 (41.9-51.1)	0.168
HSI – Remission	42.9 (40.2-49.2)	44.51 (38.7-48.4)	0.273
p-value (ΔHSI)	0.007	0.003	

Hepatosteatosis Index, HSI; change in Hepatosteatosis Index, ΔHSI.

Within-group analyses demonstrated a statistically significant reduction in HSI from the active phase to the remission period in both groups. In patients with adrenal CS, HSI decreased significantly over six months (*p* = 0.007), and a similar reduction was observed in patients with pituitary CS (*p* = 0.003).

Six-month changes in FIB-4 after remission are summarized in **Table 4**. In the active phase, median FIB-4 values were similar between adrenal and pituitary CS (0.75 [0.48–0.99] vs. 0.66 [0.47–0.97], respectively; *p* = 0.223). The proportion of patients with FIB-4 ≥ 1.3 also did not differ significantly between groups (13.3% vs. 6.4%, *p* = 0.240).

**Table 4:** Six-month change in FIB-4 after remission.

Parameter	Adrenal CS median (IQR)	Pituitary CS median (IQR)	P value
FIB-4 – Active phase	0.75 (0.48-0.99)	0.66 (0.47-0.97)	0.223
FIB-4 – 6-month remission	0.85 (0.56-1.03)	0.68 (0.51-0.92)	0.199
p-value (ΔFIB-4)	0.317	0.936	

Fibrosis-4 Index, FIB-4; ΔFIB-4, change in Fibrosis-4 index.

At six months following remission, FIB-4 remained comparable between etiological groups (0.85 [0.56–1.03] in adrenal CS vs. 0.68 [0.51–0.92] in pituitary CS; *p* = 0.199). Within-group analyses indicated no significant change in FIB-4 from the active phase to six-month remission in either adrenal (*p* = 0.317) or pituitary CS (*p* = 0.936). However, when analyzed in a group of patients with FIB-4 ≥1.3, the FIB-4 significantly decreased from the active phase to 6-month remission (median [IQR]: 1.72 [1.35–2.01] vs. 1.38 [1.14–1.46]; p = 0.010) (**Table 5****).** As shown in **Table 6**, no significant association was observed between CS etiology and FIB-4 categories defined by a cut-off value of 1.3 (χ² = 1.45, p = 0.229; Fisher’s exact test p = 0.229).

**Table 5:** Changes in FIB-4 among patients with baseline FIB-4 ≥1.3 during active disease and 6-month remission.

Parameter	Active phase Median (IQR)	6-month remission Median (IQR)	Wilcoxon signed-rank test
Pituitary CS	1.76 (1.40-2.05)	1.38 (1.18-1.50)	p = 0.018
Adrenal CS	1.68 (1.32–1.95)	1.34 (1.10–1.44)	p = 0.029
FIB-4	1.72 (1.35–2.01)	1.38 (1.14–1.46)	p *= 0.010*

Fibrosis-4 Index, FIB-4; CS, Cushing Syndrome.

**Table 6:** Association between CS Etiology and FIB-4.

CS Etiology	FIB-4 < 1.3 (n, %)	FIB-4 ≥ 1.3 (n, %)	p value
Pituitary CS	44 (93.6)	3 (6.4)	
Adrenal CS	51 (86.4)	8 (13.6)	
Overall	95 (89.6)	11 (10.4)	0.229

Fibrosis-4 Index, FIB-4; CS, Cushing Syndrome.

### 3.4 Predictors of MASLD in CS: logistic regression findings

Factors associated with MASLD were first evaluated using univariate logistic regression analysis (**Table 7**). In univariate analyses, higher BMI was significantly associated with the presence of MASLD (OR 1.25, 95% CI 1.12–1.40; *p* < 0.001), whereas increasing age was inversely associated with MASLD (OR 0.96, 95% CI 0.94–0.99; *p* = 0.016). The duration of symptoms before diagnosis showed a trend toward significance (OR 1.04, 95% CI 0.99–1.08; *p* = 0.078). Sex, CS etiology, diabetes mellitus, hypertension, DST results, midnight cortisol measurements, 24-hour UFC levels, and DHEA-S were not significantly associated with MASLD in univariate analyses (p>0.05).

**Table 7:** Univariate logistic regression analysis for factors associated with MASLD.

Variable	β (SE)	OR	95% CI	*p* value
Gender	0.347	1.41	0.51-3.88	0.500
Age	-0.037	0.964	0.94-0.99	0.016
BMI	0.224	1.25	1.12-1.40	<0.001
Cushing type (Adrenal vs Pituitary)	0.238	1.27	0.61-2.62	0.520
Diabetes mellitus	0.119	1.13	0.55-2.32	0.748
Hypertension	0.392	1.48	0.66-3.3	0.337
Duration of symptoms before diagnosis (months)	0.039	1.04	0.99-1.08	0.078
2 mg DST result, μg/dL	-0.032	0.97	0.92-1.02	0.216
1 mg DST result, μg/dL	-0.005	0.99	0.95-1.04	0.825
DHEA-S (µg/dL)	-0.003	0.997	0.994–0.999	0.095
Midnight serum cortisol (µg/dL)	-0.041	0.96	0.90-1.02	0.183
Late-night salivary cortisol (µg/dL)	-0.226	0.80	0.45-1.42	0.443
24-UFC (µg/24h)	-0.001	0.99	0.99-1.0	0.168

Body Mass Index, BMI; DST, dexamethasone suppression test; UFC, urinary free cortisol; MASLD, metabolic dysfunction–associated steatotic liver disease; DHEA-S, dehydroepiandrosterone sulfate.

Variables considered clinically relevant and/or showing an association in univariate analysis were subsequently entered into a multivariate logistic regression model (**Table 8**). In the adjusted model, higher BMI remained independently associated with MASLD (adjusted OR 1.30, 95% CI 1.15–1.49; *p* < 0.001). A longer duration of symptoms before diagnosis was also independently associated with an increased odds of MASLD (adjusted OR 1.06, 95% CI 1.005–1.114; *p* = 0.031). In contrast, increasing age remained inversely associated with MASLD after adjustment (adjusted OR 0.95, 95% CI 0.92–0.99; *p* = 0.013). Notably, the etiology of CS emerged as an independent factor in the multivariate model. Using pituitary CS as the reference category, adrenal CS was associated with higher odds of MASLD (adjusted OR 2.90, 95% CI 1.03–8.20; *p* = 0.044). Additionally, DHEA-S was identified as an independent factor inversely associated with MASLD (β = −0.05, OR = 0.997, 95% CI: 0.991–0.999, p = 0.021). Other variables, including sex, DST results, midnight serum cortisol levels, and 24-hour UFC levels, did not show an independent association with MASLD after adjustment.

**Table 8:** Multivariate logistic regression analysis for factors associated with MASLD.

Variable	β (SE)	Adjusted OR	95% CI	*p* value
BMI (kg/m²)	0.265	1.30	1.15-1.49	<0.001
2 mg DST result, μg/dL	0.005	1.00	0.93-1.09	0.903
Midnight serum cortisol (µg/dL)	0.026	1.03	0.93-1.13	0.597
24-UFC (µg/24h)	-0.001	0.99	0.99-1.00	0.275
Duration of symptoms before diagnosis	0.057	1.06	1.005-1.114	0.031
Gender	-0.239	0.79	0.23-2.70	0.704
Age (years)	-0.046	0.95	0.92-0.99	0.013
Cushing type (adrenal vs pituitary)	1.07	2.90	1.03-8.20	0.044
DHEA-S (µg/dL)	-0.05	0.997	0.991–0.999	0.021

Body Mass Index, BMI; DST, dexamethasone suppression test; UFC, urinary free cortisol; MASLD, metabolic dysfunction–associated steatotic liver disease; DHEA-S, dehydroepiandrosterone sulfate.

## 4 Discussion

In the present study, MASLD was highly prevalent among patients with CS, affecting approximately two-thirds of the cohort, underscoring the substantial hepatic metabolic burden associated with chronic hypercortisolism. At baseline, MASLD prevalence did not differ significantly between adrenal and pituitary CS. However, after adjustment for key metabolic and clinical factors, including BMI, age, and symptom duration, adrenal CS was independently associated with a higher odds of MASLD than pituitary disease. This suggests that the effect of disease etiology may be masked by shared metabolic risk factors and becomes evident only after appropriate multivariable adjustment. In this context, higher BMI and longer symptom duration before diagnosis emerged as robust independent predictors of MASLD, highlighting the importance of both metabolic load and prolonged, untreated hypercortisolism in the development of hepatic steatosis. Collectively, these findings emphasize that MASLD in CS is a multifactorial condition shaped by metabolic status, disease chronicity, and etiology-dependent factors, rather than by absolute cortisol levels. The absence of an association between MASLD and standard biochemical indices of cortisol excess in both univariate and multivariate analyses suggests that diagnostic cortisol tests reflect short-term hormonal activity and may not adequately capture the cumulative metabolic consequences of prolonged hypercortisolism that drive hepatic steatosis.

The prevalence of MASLD in our cohort (62.7%) was substantially higher than the estimated global prevalence of approximately 25%. MASLD prevalence did not differ between CS subtypes in unadjusted analyses, consistent with previous reports. Yu et al. demonstrated a significantly higher prevalence of NAFLD in patients with CS (66.1%) compared with those with mild autonomous cortisol secretion and nonfunctional adrenal incidentalomas (9). Similarly, normocortisolemic individuals with MASLD have been shown to exhibit higher cortisol concentrations than healthy controls (19), and Burcu et al. reported higher HSI and fatty liver index values in patients with mild autonomous cortisol secretion. In contrast, several studies have reported MASLD prevalence rates in CS comparable to those in the general population (10, 12). For instance, Rockall et al. evaluated hepatic steatosis using computed tomography with the liver-to-spleen ratio and demonstrated that 20% of patients with CS had hepatic steatosis (20). These discrepancies likely reflect differences in study design, diagnostic modalities, and sample size, as well as variability in metabolic risk profiles across cohorts.

Consistent with previous studies (19, 21), we observed a significant reduction in HSI from diagnosis to six months after biochemical remission, suggesting partial reversibility of hepatic steatosis following cortisol normalization. For instance, two case reports evaluating liver impairment during CS treatment described improvement in liver parameters following medical therapy for pituitary CS: in one, liver function tests (LFTs) decreased from up to 11× the upper reference limit (URL) to near-normal within 3 months, and in the other, LFTs (up to 4× URL) normalized after osilodrostat, accompanied by an early decline in FIB-4 (1.6 to 0.61 by month 4) and sustained biochemical improvement by month 9 (19). In line with these observations, no significant change in FIB-4 was observed in the overall cohort over six months, although patients with elevated baseline fibrosis risk (FIB-4 ≥1.3) experienced a significant reduction. Notably, baseline fibrosis risk was low in our cohort, with only 10.4% of patients exhibiting FIB-4 ≥1.3, which may have limited the ability to detect changes over time. This finding may also align with the proposed anti-inflammatory and antifibrotic effects of glucocorticoids on hepatic fibrogenesis (22). The divergent trajectories of HSI and FIB-4 following remission suggest that hepatic steatosis may be more rapidly reversible than fibrosis risk after cortisol normalization, with measurable improvement in fibrosis indices occurring primarily among patients with elevated baseline fibrosis risk.

In multivariable analyses, BMI, symptom duration, CS etiology, and age were independently associated with MASLD, whereas cortisol levels measured by standard diagnostic tests were not. This observation is consistent with prior studies showing no association between diagnostic cortisol measurements and hepatic steatosis (8, 20, 23). The inverse association between age and MASLD is noteworthy and may reflect survivor or referral bias, or a more pronounced metabolic response to cortisol excess in younger patients with CS.

Adrenal CS was associated with nearly threefold higher odds of MASLD compared with pituitary CS. This finding is particularly notable given that patients with pituitary CS exhibited higher cortisol and UFC levels, suggesting that factors beyond absolute cortisol burden contribute to MASLD risk. Markedly lower DHEA-S levels in adrenal CS may also contribute to this metabolic phenotype. DHEA-S has been suggested to exert protective metabolic and hepatic effects, including improved insulin sensitivity and reduced hepatic lipid accumulation. In adrenal CS, autonomous cortisol secretion suppresses ACTH, thereby reducing adrenal androgen production and lowering circulating DHEA-S levels. The relative deficiency of DHEA-S may further exacerbate insulin resistance, promote hepatic *de novo* lipogenesis, and impair lipid oxidation, thereby facilitating hepatic fat accumulation (24, 25). Consistent with this hypothesis, our multivariate analysis demonstrated that lower DHEA-S levels were independently associated with MASLD. Therefore, beyond hypercortisolemia itself, the combined effect of cortisol excess and concomitant androgen suppression may create a more unfavorable metabolic milieu in adrenal CS, potentially explaining the higher prevalence of MASLD in this subgroup.

BMI is a well-established risk factor for MASLD (2, 8), and prior studies have similarly identified BMI as an independent determinant (12). Of note, the literature contains limited research on symptom duration in MASLD in patients with CS (8). This can be considered a strength of our study. The present study elucidated that symptom duration is an independent significant factor associated with MASLD. Longer symptom duration likely reflects prolonged exposure to excess cortisol, contributing to metabolic complications such as central obesity, diabetes mellitus, and hypertension, all of which promote MASLD.

The observed associations between MASLD, metabolic burden, disease duration, and CS etiology can be further understood in light of the pleiotropic metabolic and immunomodulatory effects of glucocorticoids. Excess glucocorticoids promote lipolysis in adipose tissue, increasing free fatty acid flux to the liver and driving hepatic steatosis and insulin resistance through glucocorticoid receptor–mediated pathways. Conversely, glucocorticoids exert potent anti-inflammatory effects that may attenuate intrahepatic inflammation and delay progression from steatosis to fibrosis, potentially mediated through suppression of proinflammatory cytokines such as interleukin-6 (26–29). This dual metabolic and anti-inflammatory action may help explain the relatively low fibrosis burden observed in our cohort despite the high prevalence of MASLD.

This study has several strengths, including a relatively large, well-characterized cohort of patients with CS and the use of validated noninvasive indices (HSI and FIB-4) assessed at predefined time points, thereby allowing evaluation of changes following biochemical remission while adjusting for relevant confounders. In addition, hepatic steatosis was assessed using abdominal ultrasound, providing a pragmatic, imaging-based evaluation applicable to routine clinical practice. Several limitations should be acknowledged. The retrospective design limits causal inference and may introduce bias. HSI includes parameters such as BMI, liver enzymes, and diabetes status that may improve after CS treatment, potentially influencing the index independently of true regression of steatosis. Likewise, FIB-4 may not fully capture hepatic fibrosis in this population. Finally, the use of surrogate indices and a six-month follow-up may be insufficient, particularly for fibrosis outcomes. Prospective studies with longer follow-up and comprehensive hepatic assessment are warranted.

Collectively, our findings indicate that MASLD in CS is driven primarily by metabolic burden and disease chronicity rather than by biochemical severity at diagnosis. Higher BMI and longer symptom duration independently predicted MASLD, and adrenal etiology conferred additional risk after multivariable adjustment. These findings support routine hepatic assessment in patients with CS, particularly those with prolonged symptoms and increased metabolic risk, and underscore the need for prospective studies incorporating elastography or histological evaluation to better define long-term hepatic outcomes after remission.

## Data Availability

The raw data supporting the conclusions of this article will be made available by the authors, without undue reservation.
